# Supplementary material for: Baclofen, a GABAb receptor agonist, impairs motor learning in healthy people and changes inhibitory dynamics in motor areas
Source: Imaging Neurosci (Camb). 2025 Oct 31;3:IMAG.a.979. doi: 10.1162/IMAG.a.979 (PMC12580813; doi:10.1162/IMAG.a.979)
Supplement: Supplementary Tables [file IMAG.a.979_SupTables.pdf]

Supplementary Table 1: Minimum Reporting Standards in MRS checklist

Site (Name or Number)<br><br>1. Hardware	Nuffield Department of Clinical Neurosciences (University of Oxford)
a. Field strength [T]	3
b. Manufacturer	Siemens
c. Model	Prisma
d. RF coils	32-channel receive head coil
e. Additional hardware	N/A
2. Acquisition	
a. Pulse sequence	Semi-LASER MRSI sequence with density-weighted concentric ring trajectories (CRTs) k-space sampling<br><br>As described in<br>Steel A, Chiew M, Jezzard P, Voets NL, Plaha P, Thomas MA, Stagg CJ, Emir UE. Metabolite-cycled density-weighted concentric rings k-space trajectory (DW-CRT) enables high-resolution 1 H magnetic resonance spectroscopic imaging at 3-Tesla. Sci Rep. 2018 May 17;8(1):7792. doi: 10.1038/s41598-018-26096-y. PMID: 29773892; PMCID: PMC5958083
b. Volume of Interest (VOI) locations	Bilateral primary motor cortices and premotor cortices
c. Nominal VOI size [cm^3, mm^3]	Nominal Voxel size: 5 x 5 x 15 mm^3<br>sLASER selected VOI: 85 x 35 x 15 mm^3
d. Repetition Time (TR), Echo Time (TE) [ms, s]	TR = 1.4 s, TE = 32 ms

e. Total number of Excitations or acquisitions per spectrum	N/A for MRSI
f. Additional sequence parameters:	2D MRSI. Slab and inner volume selection achieved using semi-LASER. FOV: 240 x 240 x 15 mm^3 Matrix: 48 x 48 x 1 Nominal Voxel size: 5 x 5 x 15 mm^3 Trajectory: Density weighted concentric ring
g. Water Suppression Method	Metabolite cycling
h. Shimming Method, reference peak, and thresholds for "acceptance of shim" chosen	Vendor's own GRE-based shimming ("GRE Brain" setting)
i. Triggering or motion correction method	N/A
**3. Data analysis methods and outputs**	
a. Analysis software	In-house bash scripts (preprocessing), LCmodel (fitting and quantification)<br><br>As described in Steel A, Chiew M, Jezzard P, Voets NL, Plaha P, Thomas MA, Stagg CJ, Emir UE. Metabolite-cycled density-weighted concentric rings k-space trajectory (DW-CRT) enables high-resolution 1 H magnetic resonance spectroscopic imaging at 3-Tesla. Sci Rep. 2018 May 17;8(1):7792. doi: 10.1038/s41598-018-26096-y. PMID: 29773892; PMCID: PMC5958083.

b. Processing steps deviating from quoted reference or product	N/A
c. Output measure	Concentrations relative to total creatine (tCr)
d. Quantification references and assumptions, fitting model assumptions	Neurochemicals were quantified using LCModel with a basis set containing 26 metabolites, default LCModel macromolecules, no soft constraints on metabolites, a baseline stiffness setting (DKMTMN) of 0.25 and a chemical shift of 0.5 to 4.2ppm.
**4. Data Quality**	
a. Reported variables	LCModel measured SNR and linewidths (FWHM).
b. Data exclusion criteria	CRLB > 50%<br>SNR ratio < 40<br>GABA/tCR >1
c. Quality measures of postprocessing Model fitting	LCModel estimated CRLB.
d. Sample Spectrum	See Figure 1c

Supplementary Table 2 – summary of all repeated-measures ANOVAs conducted and post-hoc comparisons

**1. Behavioural analysis of the SRTT – 2-way repeated-measures (RM) ANOVA**		
**Learning block (S1-S11)**	**F(3.179, 41.330) = 9.261**	**p < 0.0001**
**Treatment (baclofen, placebo)**	**F(1, 13) = 6.070**	**p = 0.0286**
Learning x treatment interaction	F(4.568, 59.38) = 1.089	p = 0.374
**2. [GABA] in the left motor regions**<br>a) 2-way RM ANOVA (collapsed by ROI, [GABA] as mean of left M1 and PMC)		
Drug (baclofen, placebo)	F(1, 13) = 0.497	p = 0.493
Time (pre-, post-task)	F(1,13) = 0.923	p = 0.354
**Drug x Time interaction**	**F(1, 13) = 5.908**	**p = 0.030**
Post-hoc comparisons (Bonferroni corrected)		
**Baclofen (pre vs post task)**	**t(13) = 2.620**	**Adj p = 0.042**
Placebo (pre vs post task)	t(13) = 0.839	Adj p = 0.833
b) 3-way RM ANOVA		
**Region of interest (ROI: left M1, left PMC)**	**F (0.8725, 11.34) = 8.128**	**p = 0.018**
Drug (baclofen, placebo)	F (0.9074, 11.80) = 0.4971	p = 0.477
Time (pre-, post-task)	F (0.7701, 10.01) = 0.9226	p = 0.331
**Treatment x Task interaction**	**F(1, 13) = 5.908**	**p = 0.030**
ROI x Drug	F (1.000, 13.00) = 2.381	p = 0.147
ROI x Time	F (1.000, 13.00) = 2.207	p = 0.161
ROI x Time x Drug	F (0.7553, 9.819) = 0.5578	p = 0.425
Post-hoc comparisons (Bonferroni corrected)		
M1 baclofen (pre vs post task)	t(13) = 1.652	Adj p > 0.999
M1 placebo (pre vs post task)	t(13) = 1.796	Adj p > 0.999
PMC baclofen (pre vs post task)	t(13) = 2.624	Adj p = 0.252
PMC placebo (pre vs post task)	t(13) = 0.098	Adj p > 0.999
Pre-task baclofen (M1 vs PMC)	t(13) = 2.373	Adj p = 0.405
Pre-task placebo (M1 vs PMC)	t(13) = 0.172	Adj p > 0.999

Post-task baclofen (M1 vs PMC)	t(13) = 2.498	Adj p = 0.320
Post-task placebo (M1 vs PMC)	t(13) = 1.781	Adj p > 0.999
M1 pre-task (baclofen vs placebo)	t(13) = 2.402	Adj p = 0.384
M1 post-task (baclofen vs placebo)	t(13) = 0.709	Adj p > 0.999
PMC pre-task (baclofen vs placebo)	t(13) = 1.012	Adj p > 0.999
PMC post-task (baclofen vs placebo)	t(13) = 1.115	Adj p > 0.999

**3. [GABA] in the right motor regions**<br>a) 2-way RM ANOVA (collapsed by ROI, [GABA] as mean of right M1 and PMC; one participant's post-task placebo PMC data did not pass quality checks, so the post-task placebo M1 data was used instead of the mean)		
Drug (baclofen, placebo)	F (1.000, 13.00) = 0.05575	p = 0.817
**Time (pre-, post-task)**	**F (1.000, 13.00) = 6.012**	**p = 0.029**
Drug x Time interaction	F (1.000, 13.00) = 0.1073	p = 0.748
Post-hoc comparisons (Bonferroni corrected)		
Baclofen (pre vs post task)	t(13) = 2.028	Adj p = 0.127
Placebo (pre vs post task)	t(13) = 1.200	Adj p = 0.503
b) Mixed-effects model (REML) used due to missing data from one participant on the post-task placebo PMC measurement		
Region of interest (ROI: right M1, right PMC)	F (0.9734, 12.65) = 0.7919	p = 0.387
Drug (baclofen, placebo)	F (0.7194, 9.352) = 0.01615	p = 0.828
**Time (pre-, post-task)**	**F (0.9416, 12.24) = 5.129**	**p = 0.044**
Drug x Time interaction	F (1.000, 13.00) = 0.02606	p = 0.874
ROI x Drug	F (1.000, 13.00) = 0.2347	p = 0.636
ROI x Time	F (1.000, 13.00) = 0.5480	p = 0.472
ROI x Drug x Time	F (0.7871, 8.658) = 0.01146	p = 0.869
Post-hoc comparisons (Bonferroni corrected)		

M1 baclofen (pre vs post task)	t(13) = 1.241	Adj p = 0.961
M1 placebo (pre vs post task)	t(13) = 1.000	Adj p = 0.993
PMC baclofen (pre vs post task)	t(13) = 1.711	Adj p = 0.756
PMC placebo (pre vs post task)	t(12) = 1.377	Adj p = 0.937
Pre-task baclofen (M1 vs PMC)	t(13) = 0.436	Adj p > 0.999
Pre-task placebo (M1 vs PMC)	t(12) = 0.095	Adj p > 0.999
Post-task baclofen (M1 vs PMC)	t(13) = 1.034	Adj p = 0.990
Post-task placebo (M1 vs PMC)	t(12) = 0.566	Adj p > 0.999
M1 pre-task (baclofen vs placebo)	t(13) = 0.257	Adj p > 0.999
M1 post-task (baclofen vs placebo)	t(13) = 0.012	Adj p > 0.999
PMC pre-task (baclofen vs placebo)	t(12) = 0.633	Adj p > 0.999
PMC post-task (baclofen vs placebo)	t(12) = 0.110	Adj p > 0.999

**4. [Glu] in the left motor regions**<br>a) 2-way RM ANOVA (collapsed by ROI, [Glu] as mean of left M1 and PMC)		
Drug (baclofen, placebo)	F (1.000, 13.00) = 0.3741	p = 0.551
**Time (pre-, post-task)**	**F (1.000, 13.00) = 8.174**	**p = 0.013**
Drug x Time interaction	F (1.000, 13.00) = 0.5785	p = 0.461
Post-hoc comparisons (Bonferroni corrected)		
Baclofen (pre vs post task)	t(13) = 1.568	Adj p = 0.282
Placebo (pre vs post task)	t(13) = 2.414	Adj p = 0.063
b) 3-way RM ANOVA		
Region of interest (ROI: left M1, left PMC)	F (0.8534, 11.09) = 39.08	**p < 0.001**
Drug (baclofen, placebo)	F (0.9607, 12.49) = 0.3741	p = 0.543
**Time (pre-, post-task)**	**F (0.8398, 10.92) = 8.174**	**p = 0.019**
Drug x Time interaction	F (1.000, 13.00) = 0.5785	p = 0.461
ROI x Drug	F (1.000, 13.00) = 0.4197	p = 0.528
ROI x Time	F (1.000, 13.00) = 1.782	p = 0.205
ROI x Drug x Time	F (0.7328, 9.527) = 0.4549	p = 0.457

Post-hoc comparisons (Bonferroni corrected)		
M1 baclofen (pre vs post task)	t(13) = 1.325	Adj p > 0.999
**M1 placebo (pre vs post task)**	**t(13) = 4.079**	**Adj p = 0.016**
PMC baclofen (pre vs post task)	t(13) = 1.325	Adj p > 0.999
PMC placebo (pre vs post task)	t(13) = 0.843	Adj p > 0.999
**Pre-task baclofen (M1 vs PMC)**	**t(13) = 4.729**	**Adj p = 0.005**
**Pre-task placebo (M1 vs PMC)**	**t(13) = 5.117**	**Adj p = 0.002**
Post-task baclofen (M1 vs PMC)	t(13) = 3.321	Adj p = 0.066
Post-task placebo (M1 vs PMC)	t(13) = 2.549	Adj p = 0.291
M1 pre-task (baclofen vs placebo)	t(13) = 0.541	Adj p > 0.999
M1 post-task (baclofen vs placebo)	t(13) = 0.925	Adj p > 0.999
PMC pre-task (baclofen vs placebo)	t(13) = 0.627	Adj p > 0.999
PMC post-task (baclofen vs placebo)	t(13) = 0.639	Adj p > 0.999

13

6. **Mood components**<br>a) **Alertness**: 2-way RM ANOVA		
Drug (baclofen, placebo)	F (1.000, 14.00) = 4.164	p = 0.061
**Time (baseline, 1h, 2.5h)**	**F (1.775, 24.86) = 11.18**	**p = 0.0005**
Drug x Time interaction	F (1.348, 18.88) = 2.389	p = 0.132
Post-hoc comparisons (Bonferroni corrected)		
Baseline (baclofen vs placebo)	t(14) = 1.791	Adj p = 0.285
1h (baclofen vs placebo)	t(14) = 0.488	Adj p > 0.999
2h (baclofen vs placebo)	t(14) = 2.199	Adj p = 0.136
b) **Contentedness**: 2-way RM ANOVA		
**Drug (baclofen, placebo)**	**F (1.000, 14.00) = 5.142**	**p = 0.040**
Time (baseline, 1h, 2.5h)	F (1.362, 19.07) = 0.3845	p = 0.609
Drug x Time interaction	F (1.945, 27.23) = 0.8992	p = 0.416
Post-hoc comparisons (Bonferroni corrected)		
Baseline (baclofen vs placebo)	t(14) = 1.980	Adj p = 0.203
1h (baclofen vs placebo)	t(14) = 1.992	Adj p = 0.199

2h (baclofen vs placebo)	t(14) = 0.566	Adj p > 0.999
c) **Calmness**: 2-way RM ANOVA		
Drug (baclofen, placebo)	F (1.000, 14.00) = 0.6529	p = 0.433
Time (baseline, 1h, 2.5h)	F (1.556, 21.78) = 0.1628	p = 0.797
Drug x Time interaction	F (1.310, 18.34) = 0.7728	p = 0.425
Post-hoc comparisons (Bonferroni corrected)		
Baseline (baclofen vs placebo)	t(14) = 0.165	Adj p > 0.999
1h (baclofen vs placebo)	t(14) = 1.586	Adj p = 0.405
2h (baclofen vs placebo)	t(14) = 0.610	Adj p > 0.999

**7. Working memory tests**<br>a) Total errors on the spatial working memory task (Mixed-effects model (REML) used due to missing data; within-subject main factors of drug and difficulty)		
Drug (baclofen, placebo)	F(1, 17) = 0.335	p = 0.570
**Difficulty (4, 6, 8 and 12 blocks)**	**F (1.084, 18.43) = 65.66**	**p < 0.0001**
Drug x difficulty interaction	F (1.181, 17.710) = 0.214	p = 0.689
b) Percentage of correct responses on the pattern recognition memory task (Mixed-effects model (REML) used due to missing data; within-subject main factors of drug and time)		
Drug (baclofen, placebo)	F (1, 17) = 1.294	p = 0.271
Time (immediate, delayed)	F (1, 17) = 2.766	p = 0.115
Drug x time interaction	F (1, 15) = 0.629	p = 0.440
c) Response times on the pattern recognition memory task (Mixed-effects model (REML) used due to missing data; within-subject main factors of drug and time)		
Drug (baclofen, placebo)	F (1, 17) = 1.540	p = 0.232
**Time (immediate, delayed)**	**F (1, 17) = 15.90**	**p = 0.001**
Drug x time interaction	F (1, 15) = 1.131	p = 0.304

d) Maximum length achieved on the spatial span task (2-way repeated-measures ANOVA with within-subject factors of drug and direction)		
Drug (baclofen, placebo)	F (1, 17) = 2.227	p = 0.154
Direction (forward, reverse)	F (1, 17) = 0.979	p = 0.336
Drug x time interaction	F (1, 17) = 0.308	p = 0.586
e) Number of errors on the spatial span task (2-way repeated-measures ANOVA with within-subject factors of drug and direction)		
Drug (baclofen, placebo)	F (1, 17) = 0.221	p = 0.644
Direction (forward, reverse)	F (1, 17) = 0.086	p = 0.773
Drug x time interaction	F (1, 17) = 0.087	p = 0.772
