# Supplementary material for: Baclofen, a GABAb receptor agonist, impairs motor learning in healthy people and changes inhibitory dynamics in motor areas
Source: Imaging Neurosci (Camb). 2025 Oct 31;3:IMAG.a.979. doi: 10.1162/IMAG.a.979 (PMC12580813; doi:10.1162/IMAG.a.979)
Supplement: Supplementary Figures [file IMAG.a.979_SupFig1.pdf]

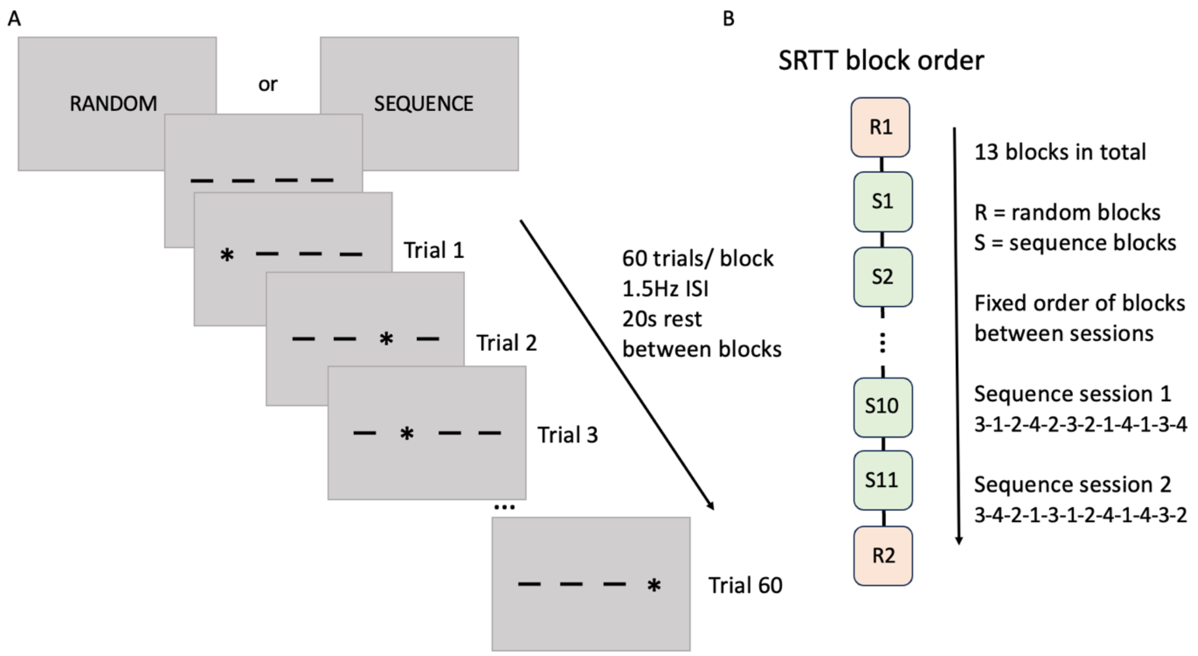

Supplementary Fig. 1 – Serial reaction time task (SRTT) design. (A) Each block (either random or sequence) started with an instruction screen stating whether the following block will be random or the sequence. After that, 60 trials were presented at an interstimulus frequency of 1.5Hz, with one of the 4 dashes turning into an asterisk indicating which button should be pressed next. If the participant pressed the correct button, the asterisk would turn back into a dash until the next trial would start. If the participant did not press the correct (or any) button, the asterisk would remain present until the next trial began. The 60 trials in each block would be completed in 40 seconds, followed by 20 seconds of rest between blocks. (B) In each session, there were 13 blocks of the SRTT task presented in a fixed order: 11 sequence blocks flanked by one random block. The sequences were different between the 2 sessions, but included the same number of button presses for each of the four buttons.
